# Citrus flavonoid supplement enhances glycemic and metabolic control in prediabetic patients on metformin: a randomized controlled trial

**DOI:** 10.3389/fnut.2025.1639901

**Published:** 2025-08-19

**Authors:** Thais Cesar, Maria Rita Oliveira, Valeria Sandrim, Adriana Mendes, Ricardo Bruder, Rogerio Oliveira, Katia Sivieri, Dragan Milenkovic

**Affiliations:** ^1^Graduate Program in Food, Nutrition, and Food Engineering, São Paulo State University (UNESP), Araraquara, Brazil; ^2^Department of Nutrition, Institute of Biosciences, São Paulo State University (UNESP), Botucatu, Brazil; ^3^Department of Biophysics and Pharmacology, Institute of Biosciences, São Paulo State University (UNESP), Botucatu, Brazil; ^4^Department of Internal Medicine, Institute of Biosciences, São Paulo State University (UNESP), Botucatu, Brazil; ^5^Department de Biostatistics, Institute of Biosciences, São Paulo State University (UNESP), Botucatu, Brazil; ^6^Graduate Program in Biotechnology in Regenerative Medicine and Medicinal Chemistry Food, Department of Food and Nutrition, Faculty of Pharmaceutical Sciences, University of Araraquara (UNIARA), Araraquara, Brazil; ^7^Plants for Human Health Institute, North Carolina State University, Kannapolis, NC, United States

**Keywords:** citrus flavonoids, nutraceutical, metformin, GLP-1, inflammatory biomarkers, antioxidant status, prediabetes

## Abstract

**Background and objective:**

Combining natural compounds with conventional drugs is an emerging strategy to improve the management of type 2 diabetes and its precursor, prediabetes. While metformin effectively lowers blood glucose and improves insulin sensitivity, it may cause side effects or lose efficacy over time. Natural agents, particularly polyphenols, are being explored as adjunct therapies to enhance glycemic control, mitigate adverse effects, and slow disease progression. This study evaluated the efficacy of a citrus bioflavonoid-based nutraceutical as an adjunct to metformin therapy in prediabetic individuals, with a focus on metabolic, inflammatory, oxidative, hormonal, and nutritional-clinical outcomes.

**Methods:**

In this 12-week randomized, double-blind, placebo-controlled trial, participants received either metformin plus the citrus flavonoid supplement (250 mg/day) or metformin plus placebo.

**Results:**

At the end of the intervention, the nutraceutical group demonstrated improved postprandial glucose metabolism, including a 5% reduction in 2-h OGTT glucose and preservation of active GLP-1 levels. In contrast, the placebo group exhibited a decline in GLP-1 and increased insulin resistance. Supplementation also resulted in a 12% reduction in TNF-*α*, a 7.5% increase in plasma antioxidant capacity (FRAP), and modest but significant decreases in body weight, fat mass, and BMI (all *p* ≤ 0.05). Additionally, systolic blood pressure was reduced by 4%, potentially associated with improved antioxidant status and higher dietary potassium intake.

**Conclusion:**

These findings suggest that citrus flavonoids may serve as a safe and effective nutritional adjunct to metformin in the early management of prediabetes. Benefits include improved postprandial glycemia, maintenance of GLP-1 levels, reduced inflammation and oxidative stress, and modest improvements in body composition and blood pressure. Further long-term studies are warranted to confirm these outcomes and elucidate underlying mechanisms.

**Clinical trial registration:**

https://clinicaltrials.gov/, identifier NCT06005142.

## Introduction

Metformin, the standard first-line treatment for type 2 diabetes (T2D), prediabetes, and metabolic syndrome, is associated with numerous benefits. It lowers both fasting and postprandial glucose levels, reduces HbA1c, improves peripheral glucose uptake, enhances insulin sensitivity, and inhibits hepatic gluconeogenesis (1). A key mechanism is its ability to increase circulating levels of glucagon-like peptide-1 (GLP-1), an incretin hormone that promotes insulin secretion, suppresses glucagon release, and slows gastric emptying (2). Despite its well-documented benefits, some individuals exhibit a suboptimal glycemic response or experience gastrointestinal side effects, which can limit metformin’s long-term use. Additionally, its efficacy may diminish over time, often requiring the addition of other hypoglycemic agents to maintain glycemic control (3).

Certain polyphenols, such as citrus flavonoids, have demonstrated notable antidiabetic effects by modulating GLP-1 secretion and activity or by inhibiting dipeptidyl peptidase-4 (DPP-4), thereby prolonging GLP-1 lifespan and activity. Eriocitrin and hesperidin have shown antihyperglycemic, antioxidant, and anti-inflammatory properties in both preclinical and clinical studies (4). Additionally, naringenin has been shown to inhibit DPP-4 activity and extend the half-life of GLP-1 (5). In previous human studies, a citrus flavonoid-based nutraceutical containing eriocitrin, hesperidin, and naringin was shown to reduce intermediate hyperglycemia after 12 weeks of daily supplementation in both prediabetic and diabetic patients (6, 7). Several mechanisms may underlie the nutraceutical’s effects, including improved metabolic health and reduced risk of disease progression—both associated with increased antioxidant capacity and modulation of inflammatory biomarkers. Additionally, an average increase of 17% in circulating GLP-1 levels was observed, which is likely linked to the hypoglycemic effect of this supplement (7).

After oral intake, citrus flavonoids remain largely intact through the gastrointestinal tract until reaching the colon, where they are deglycosylated by the intestinal microbiota. The resulting aglycone metabolites are absorbed by enterocytes, while remaining glycosides are fermented into short-chain fatty acids (SCFAs) (8). Once absorbed, aglycones such as eriodictyol, hesperetin, and naringenin are metabolized locally or transported via the portal vein to the liver, where they undergo extensive phase II metabolism into glucuronide and sulfate conjugates. These are released into systemic circulation and contribute to the observed biological effects (9, 10).

Consequently, there is growing interest in natural add-on therapies—particularly polyphenols—which may enhance metformin’s efficacy and slow diabetes progression in early-stage metabolic dysfunction (4, 5, 11). Recent strategies emphasize combining natural agents with conventional antidiabetic drugs such as metformin to optimize glycemic control. These phytochemical adjuvants may offer benefits over monotherapy, including improved glycemic regulation, reduced HbA1c levels, and fewer side effects (12–14).

Recent systematic reviews have reinforced the therapeutic potential of citrus flavonoids—particularly eriocitrin, hesperidin, and naringenin—in modulating glycemic control through antioxidant, anti-inflammatory, and insulin-sensitizing actions (15–17). These compounds have been shown to influence key metabolic pathways, including glucagon-like peptide-1 (GLP-1) secretion, dipeptidyl peptidase-4 (DPP-4) inhibition, and AMP-activated protein kinase (AMPK) activation—mechanisms directly relevant to managing early metabolic dysfunction. Clinical evidence supports their effectiveness in improving fasting glucose, postprandial glycemia, and insulin resistance, particularly when used in combination with standard pharmacologic agents such as metformin (6, 16, 17).

Pharmacokinetic studies indicate that citrus flavonoids largely bypass absorption in the upper gastrointestinal tract, reaching the colon where they are deglycosylated by the gut microbiota. The resulting aglycone metabolites are absorbed by enterocytes, while residual glycosides undergo fermentation to produce short-chain fatty acids (SCFAs) (18). In this context, citrus flavonoids may provide valuable complementary effects in populations with early metabolic impairment. The present study builds on this evidence by evaluating the clinical efficacy of a citrus flavonoid supplement as an adjunct to metformin in prediabetic individuals, using a randomized, placebo-controlled, crossover design.

Based on these findings, the central hypothesis of this study is that a citrus flavonoid-based nutraceutical, when combined with metformin, will enhance glycemic control and insulin sensitivity beyond the effects of biguanide monotherapy. Additionally, the nutraceutical-metformin combination is expected to improve glucose metabolism via GLP-1 modulation and reduce adverse effects linked to long-term metformin use. Therefore, our primary goal was to evaluate the effects of the citrus flavonoid supplement (250 mg/day) combined with metformin on glycemic control, insulin resistance, and clinical and metabolic biomarkers.

The primary outcome of the study was fasting plasma glucose (FPG) under nutraceutical add-on therapy in individuals taking metformin. Secondary outcomes included the oral glucose tolerance test (OGTT), glycated hemoglobin (HbA1c), homeostatic model assessment for insulin resistance (HOMA-IR), and lipid profile. A panel of metabolic hormones and peptides related to energy balance and glucose regulation was assessed, including GLP-1, peptide YY, insulin, C-peptide, glucagon, leptin, pancreatic polypeptide (PP), ghrelin, secretin, and amylin. Inflammatory biomarkers (hsCRP, MCP-1, IFN-*γ*, IL-6, and TNF-*α*), total antioxidant capacity (FRAP), and liver enzymes (ALP, γ-GT, AST, ALT) were also measured. Additional evaluations included pancreatic and renal function markers (amylase, lipase, urea, uric acid, and creatinine), body composition (BMI, fat mass, lean mass, circumferences), blood pressure, and dietary nutrient intake.

## Materials and methods

### Participants

Participants were recruited from the outpatient clinic of the *Hospital de Clinicas de Botucatu* (UNESP), Botucatu, SP, Brazil. Eligible individuals were men and women aged 18–65 years with prediabetes, defined according to the American Diabetes Association (ADA, 2017) criteria: fasting plasma glucose (FPG) of 100–125 mg/dL (5.6–6.9 mmol/L), 2-h plasma glucose (2-h PG) of 140–199 mg/dL (7.8–11.0 mmol/L) following a 75-g oral glucose tolerance test (OGTT), or glycated hemoglobin (HbA1c) of 5.7–6.4%. All participants had been on stable metformin monotherapy (1,000 mg/day) for at least 2 weeks before study initiation.

Exclusion criteria included physiological or pathological conditions affecting glucose homeostasis; use of additional antihyperglycemic agents, lipid-lowering drugs, anorectic medications, or dietary supplements containing bioflavonoids, vitamins, minerals, probiotics, or prebiotics; engagement in high-intensity physical activity (>10 h/week); and the presence of chronic gastrointestinal disorders.

Participants were monitored monthly for physical activity (structured interviews), dietary intake (3-day food records), and anthropometric parameters (standardized assessments).

The study was approved by the Human Research Ethics Committee of UNESP (protocol number 5.773.022/2022), conducted in accordance with the Declaration of Helsinki, and registered at ClinicalTrials.gov (Identifier: NCT06005142, https://clinicaltrials.gov/). Written informed consent was obtained from all participants.

### Study design and participants

This was a 26-week (6.5-month), double-blind, randomized, placebo-controlled, crossover clinical trial evaluating the effects of a citrus flavonoid-based nutraceutical as an add-on to metformin therapy in prediabetic patients. The intervention included two 12-week treatment periods separated by a 2-week washout phase. All participants were treated with metformin (1,000 mg/day) throughout the study. The trial design allowed for the assessment of both immediate and carryover effects of the citrus flavonoid supplement compared to placebo.

A 4-week run-in period preceded randomization and included clinical screening and nutritional assessments (anthropometric measurements, bioelectrical impedance analysis [BIA], dietary recalls, and oral glucose tolerance test [OGTT]). Eligible participants were identified by a physician and a registered nutritionist and were contacted by phone or email to receive study information. Those who met the inclusion criteria and provided written informed consent underwent baseline assessments prior to randomization.

Randomization was performed by an independent biostatistician using a computer-generated sequence. Group assignments were concealed in sequentially numbered, opaque, sealed envelopes, and allocation was revealed only after participant enrollment. Both investigators and participants remained blinded until the completion of data analysis.

**Figure 1:**
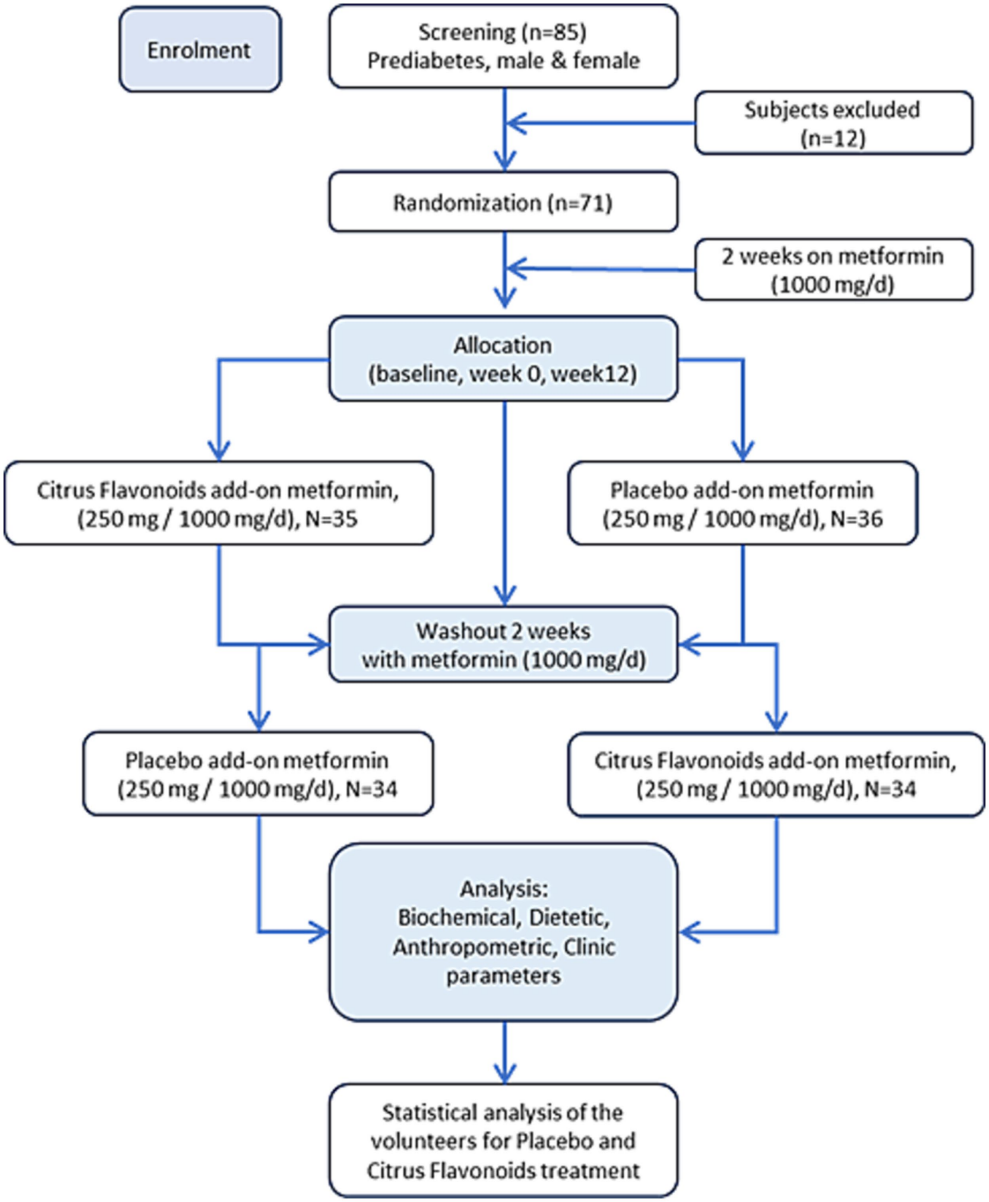
Consort flow diagram of citrus flavonoids or placebo add-on metformin trial.

Participants were randomized into two groups (Figure 1):

- **Group A (*n* = 35):** Received the citrus flavonoid-based nutraceutical (250 mg/day) plus metformin (1,000 mg/day) for the first 12 weeks, followed by a 2-week washout period, and then received placebo (250 mg/day) plus metformin (1,000 mg/day) for the subsequent 12 weeks.
- **Group B (*n* = 36):** Received placebo (250 mg/day) plus metformin (1,000 mg/day) for the first 12 weeks, followed by a 2-week washout period, and then received the citrus flavonoid supplement (250 mg/day) plus metformin (1,000 mg/day) for the final 12 weeks.

All supplements were administered once daily with the evening meal. At each monthly visit, participants returned their capsule bottles—whether empty, partially full, or with remaining capsules—to monitor adherence. Participants missing more than three capsules per month were withdrawn from the study and excluded from further analysis.

Participants were instructed to maintain their usual diet and physical activity throughout the study. To assess dietary consistency, they completed 3-day food records at weeks 0, 12, 14, and 26. The study was conducted in accordance with the CONSORT 2010 guidelines. The 2-week washout period was considered sufficient to minimize residual effects, and no significant carryover effects were observed, confirming the validity of the crossover design.

### Intervention: citrus flavonoid supplementation with metformin

The intervention product—a standardized citrus flavonoid-based nutraceutical—was provided by Ingredients by Nature (commercial name Eriomin^®^-Montclair, CA, United States). Each capsule contained 250 mg of a citrus bioflavonoid blend with 70% eriocitrin (175 mg), 5% hesperidin (12.5 mg), 4% naringin (10 mg), 1% didymin (2.5 mg), and 20% citrus-derived fibrous material (suberin, cutin, lignin, pectin, and cellulose). This dosage was based on prior clinical findings showing 200 mg daily had comparable efficacy to higher doses. Placebo capsules (microcrystalline corn starch) matched the active supplement in appearance. All capsules were manufactured under GMP conditions and dispensed biweekly. Participants were instructed to take one capsule of metformin (1,000 mg/day) and one capsule of the citrus flavonoid supplement or placebo (250 mg/day) daily after dinner with water. To ensure adherence, both the supplements and metformin tablets were dispensed biweekly.

### Clinical and nutritional assessments

Participants underwent clinical and nutritional evaluations conducted by a board-certified metabolic physician (MD) and a registered nutritionist. Assessments included body composition measurements—weight, height, circumferences, and bioelectrical impedance analysis (BIA)—as well as dietary evaluations. Prior to each assessment, participants were instructed to fast for 8–12 h, avoid alcohol consumption, wear light clothing, and refrain from intense physical activity to ensure accurate anthropometric measurements.

### Blood collection for biomarker analysis

**Figure 2:**
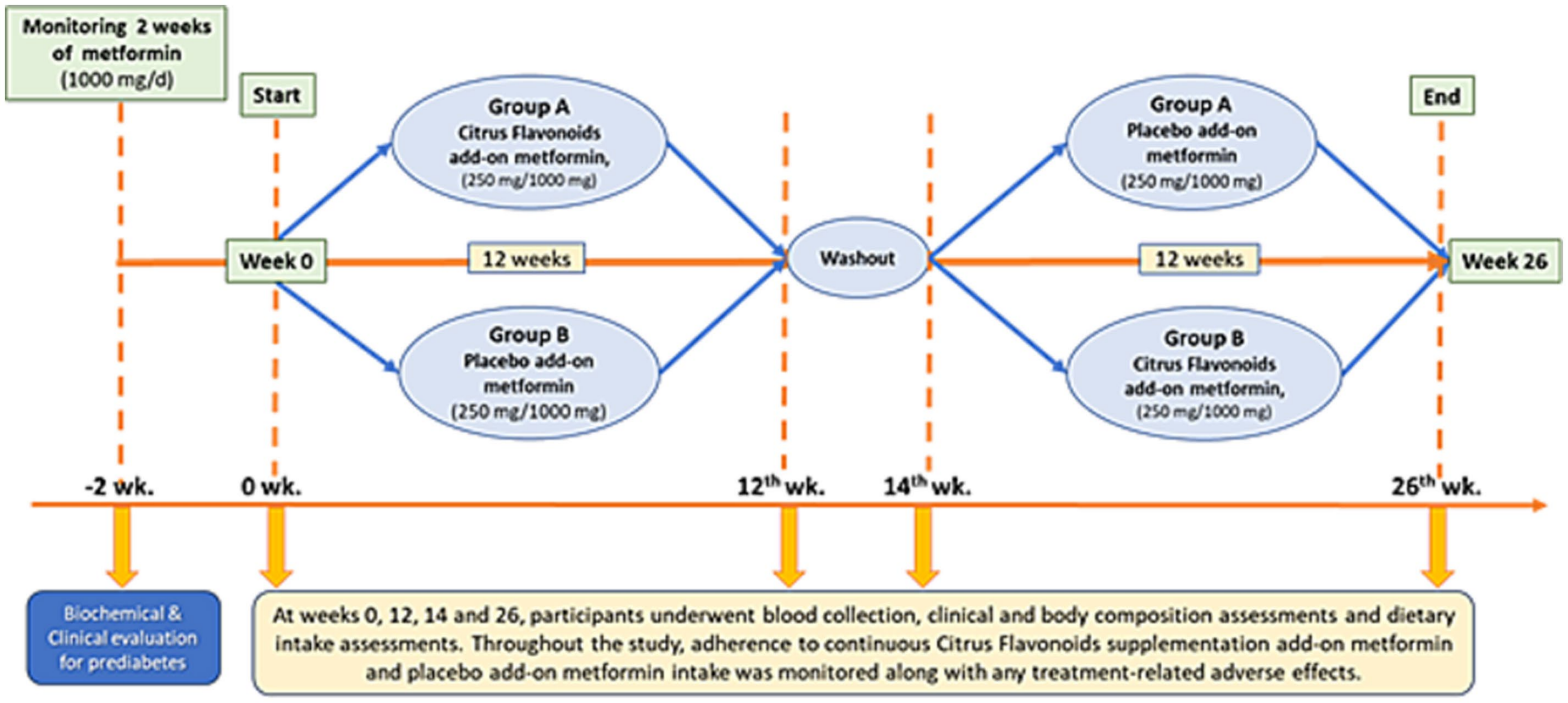
A 26-week randomized, double-blind, placebo-controlled, crossover clinical trial was conducted, consisting of two 12-week treatment periods separated by a 2-week washout phase. Participants (*n* = 68) were randomly assigned to one of two groups. Group A received citrus flavonoids as an add-on to metformin for the first 12 weeks, followed by a 2-week washout, and then received placebo as an add-on to metformin for the remaining 12 weeks. Group B followed the reverse sequence, receiving placebo first and then citrus flavonoids, both as add-on therapies to metformin.

A total of seven blood samples (30 mL per collection) were obtained from each participant throughout the study. The first sample was collected during the 2-week run-in phase to confirm prediabetes status. Subsequent collections occurred at weeks 0, 12, 14, and 26 (Figure 2). All blood draws were performed at BIOTEST Clinical Analysis Laboratory Ltd. (Botucatu, SP, Brazil) by certified phlebotomists. Plasma and serum samples were immediately processed and stored at −80°C until analysis.

The following biomarkers were analyzed using validated protocols:

- **Glucose Metabolism:** Fasting plasma glucose (FPG) and oral glucose tolerance test (OGTT) were assessed by enzymatic colorimetric methods (Labtest, Brazil); glycated hemoglobin (HbA1c) by immunoturbidimetry (Labtest, Brazil); insulin by electrochemiluminescence (Roche, United States); and insulin resistance was estimated using the HOMA-IR index.
- **Lipid Profile:** Total cholesterol and triglycerides were measured using the Trinder^®^ enzymatic method; high-density lipoprotein cholesterol (HDL-C) by selective inhibition (Labtest, Brazil).
- **Organ Function Markers:** Alkaline phosphatase (ALP), gamma-glutamyl transferase (*γ*-GT), aspartate aminotransferase (AST), alanine aminotransferase (ALT), amylase, lipase, urea, uric acid, and creatinine were analyzed using Labtest diagnostic kits (Brazil).
- **Hormonal and Inflammatory Markers:** Glucagon-like peptide-1 (GLP-1), peptide YY (PYY), insulin, C-peptide, glucagon, leptin, pancreatic polypeptide (PP), ghrelin, monocyte chemoattractant protein-1 (MCP-1), interferon-gamma (IFN-γ), interleukin-6 (IL-6), and tumor necrosis factor-alpha (TNF-*α*) were quantified using Luminex Milliplex^®^ multiplex assays (Millipore, United States). High-sensitivity C-reactive protein (hsCRP) was measured via nephelometry (Dade Behring, United States).
- **Oxidative Stress:** Total antioxidant capacity was determined using the ferric reducing ability of plasma (FRAP) assay, with absorbance measured at 593 nm (Synergy 4, Biotek).

### Compliance, adverse events, and safety

Participants were monitored monthly for supplement adherence, side effects, and adverse events. Compliance was evaluated through capsule counts and self-reported adherence. Among the total participants, the citrus flavonoid supplement used as an add-on to metformin for 12 weeks was well tolerated. No major adverse events were reported, and there were no significant changes in hepatic safety markers (AST, ALT, ALP, *γ*-GT), indicating a favorable safety profile.

### Statistical analysis

Sample size was based on previous studies of citrus flavonoid nutraceuticals (7), with 80% power, a 5% significance level, and a 30% anticipated dropout rate. Data were analyzed using repeated-measures ANCOVA, with treatment, period, and sequence as fixed effects, and washout duration as a covariate. Normality and homoscedasticity were evaluated using histograms, Q–Q plots, scatter plots, and the Kolmogorov–Smirnov test. Outliers were excluded when model assumptions were violated.

*Post hoc* analyses were performed using orthogonal contrasts. Missing data were handled using the last observation carried forward (LOCF) method for participants who completed at least one post-baseline assessment. All analyses were performed using SAS 9.4 (SAS Institute, Cary, NC, United States), and plots were generated in RStudio (v2023.12.0.369, Posit Team, United States). A two-sided *p*-value < 0.05 was considered statistically significant.

## Results

### General characteristics of the volunteers

Of the 85 individuals screened, 71 men and women (mean age: 53.2 years; mean BMI: 31.8 kg/m^2^) were randomized and included in the final data analysis. Baseline characteristics and metabolic biomarkers were comparable between participants during their time in Group A and Group B of the crossover intervention (Table 1). Individual measurements of fasting plasma glucose (FPG), 2-h plasma glucose (2-h PG), and glycated hemoglobin (HbA1c) were all within the prediabetes diagnostic criteria as defined by the American Diabetes Association (ADA). In addition, 55% of participants had HOMA-IR values ≥ 2.71, indicating that a majority of the cohort was insulin resistant at baseline.

**Table 1:** Baseline characteristics of patients randomized to receive citrus flavonoids add-on to metformin followed by placebo (Group A), or placebo followed by citrus flavonoids (Group B) after washout period.

Baseline characteristics		Group A	Group B	*P* value
*n*	71	35	36	
Sex^a^	Female	24 (71%)	22 (65%)	0.46
	Male	10 (29%)	12 (35%)	
Age^b^	Mean (DP)	54.0 (8.5)	50.1 (9.4)	0.10
	Median (Min–Max)	56.5 (48–60)	49.0 (44–58)	
BMI^b^	Mean (DP)	31.3 (6.3)	31.8 (6.2)	0.34
	Median (Min–Max)	31.0 (27.7–33.0)	30.5 (27.5–34.5)	
FPG^b^	Mean (DP)	107 (12.3)	104 (9.7)	0.30
	Median (Min–Max)	105 (80–129)	104 (83–124)	
2-h PG^b^	Mean (DP)	153 (49)	155 (43)	0.87
	Median (Min–Max)	145 (76–262)	140 (88–256)	
HbA1c^b^	Mean (DP)	5.78 (0.32)	5.75 (0.22)	0.48
	Median (Min–Max)	5.80 (5.2–6.6)	5.6 (5.0–6.0)	

Statistics among variables: ^a^ Chi-square test, and ^b^ Student’s *t*-test: BMI (Body Mass Index), FPG (Fasting Plasma Glucose), Glucose 120 min (after 75 g Glucose); HbA1C (Glycated Hemoglobin).

### Anthropometric and dietary outcomes

After completing the full treatment period, participants who adhered to the study protocol demonstrated generally stable anthropometric and dietary parameters (Table 2). Among the 68 individuals analyzed, most measures remained unchanged in both the Citrus Flavonoids and Placebo groups. However, the group receiving Citrus Flavonoids as an add-on to metformin showed modest but statistically significant reductions in body weight, BMI, and body fat mass after 12 weeks. In contrast, the Placebo plus metformin group did not exhibit significant changes in most of anthropometric parameters (Table 2). Notably, the Placebo group experienced a significant increase in neck diameter, a recognized marker of upper-body subcutaneous fat accumulation, which is metabolically active and closely linked to insulin resistance, a defining feature of prediabetes (Table 2).

**Table 2:** Anthropometric characteristics and dietary consumption of prediabetic patients treated with metformin combined with citrus flavonoids or placebo for 12 weeks.

Treatment with metformin add-on	Citrus flavonoids (*n* = 68)		Placebo (*n* = 68)		*P* value
	Week 0	Week 12	Week 0	Week 12	
	Mean (SD)	Mean (SD)	Mean (SD)	Mean (SD)	
Anthropometric characteristics					
Weight, kg	**87.2 (18.5)** ^**a**^	**86.6 (18.8)** ^**b**^	86.6 (18.7)	86.5 (18.9)	0.10
BMI kg/m^2^	**31.9 (6.3)** ^**a**^	**31.7 (6.5)** ^**b**^	31.7 (6.4)	31.7 (6.5)	0.10
Waist circumference, cm	104 (13)	104 (13)	104 (13)	104 (13)	0.57
Waist/height ratio	0.63 (0.08)	0.63 (0.08)	0.63 (0.08)	0.63 (0.08)	0.55
Abdomen diameter, cm	25.4 (3.5)	25.2 (3.58)	25.3 (3.4)	25.1 (3.5)	0.46
Neck diameter, cm	38.9 (3.9)	38.8 (3.7)	**38.8 (3.6)** ^**c**^	**39.1 (3.7)** ^**d**^	0.12
Lean mass, cm	34.3 (5.3)	34.6 (5.4)	34.6 (5.7)	34.7 (5.4)	0.90
% body fat	**38.5 (8.9)** ^**a**^	**38.0 (9.3)** ^**b**^	38.3 (9.4)	37.9 (9.0)	0.59
BMR, kcal	1,520 (258)	1,518 (259)	1,519 (258)	1,519 (254)	0.57
Dietary consumption					
Energy, kcal/d	1975 (798)	1925 (602)	1959 (696)	1845 (770)	0.34
Carbohydrate, g/d	248 (124)	240 (82)	251 (118)	232 (132)	0.26
Protein, g/d	86.8 (35.8)	83.2 (36.9)	95.6 (53.2)	88.5 (38.4)	0.88
Total fat, g	71.3 (35.8)	63.8 (28.5)	63.5 (35.4)	61.8 (38.8)	0.54
Saturated fat, g/d	22.2 (12.7)	20.7 (12.4)	**20.7 (12.1)** ^**c**^	**21.0 (14.1)** ^**d**^	0.35
Dietary fiber, g/d	21.4 (10.7)	21.1 (12.7)	24.8 (12.9)	19.7 (11.1)	0.21
Vitamin C, mg/d	147 (120)	115 (132)	147 (128)	97.0 (107)	0.67
Folate, ug/d	345 (188)	273 (125)	351 (224)	262 (111)	0.89
Sodium, g/d	2.2 (1.6)	1.8 (1.1)	1.9 (0.9)	1.7 (1.1)	0.91
Potassium, g/d	**2.2 (0.8)** ^**a**^	**2.4 (1.1)** ^**b**^	2.7 (2.6)	2.2 (1.0)	0.03*

Statistical intragroup differences between weeks 0 and 12 are indicated by different letters (“a” vs. “b” for the citrus flavonoids group and “c” vs. “d” for the placebo group) (*p* ≤ 0.05). Intergroup differences between the citrus flavonoids and placebo groups are indicated by an asterisk (*) (*p* ≤ 0.05). Statistically significant differences are indicated by the values highlighted in bold.

Dietary intake, including total energy, macronutrients, and selected micronutrients, was largely comparable between groups. However, a 1.5% higher saturated fat intake was observed in the Placebo group at week 12. Intake of vitamin C, folate, and sodium was similar across both groups. Interestingly, participants receiving Citrus Flavonoids had an 8.3% higher potassium intake, which may reflect improved dietary quality or supplement-related effects (Table 2).

### Postprandial glucose response, biochemical metabolism, and blood pressure

Postprandial glucose responses following a 75 g oral glucose load revealed significant differences between groups. In the Placebo group, blood glucose increased by 5% at 60 min and 7% at 120 min post-load, indicating impaired glucose tolerance. In contrast, the Citrus Flavonoids group did not exhibit increases at either time point, suggesting a protective effect of the supplement. Notably, the 120-min glucose level was significantly higher in the Placebo group, whereas it remained stable in the Citrus Flavonoids group, reinforcing the beneficial modulation of postprandial glycemia (Table 3).

**Table 3:** Biochemical and artery blood pressure evaluation of prediabetic patients treated with metformin combined with citrus flavonoids or placebo for 12 weeks.

Treatment with metformin add-on	Citrus flavonoids (*n* = 68)		Placebo (*n* = 68)		*P* value
	Week 0	Week 12	Week 0	Week12	
	Mean (SD)	Mean (SD)	Mean (SD)	Mean (SD)	
Biochemical parameters					
Glucose after OGTT, mg/dL					
0 min	106 (11)	105 (13)	104 (11)	105 (11)	0.49
30 min	183 (30)	185 (32)	185 (35)	189 (33)	0.45
60 min	190 (46)	197 (44)	190 (46)^c^	200 (48)^d^	0.70
120 min	157 (46)	149 (44)	145 (51)^c^	155 (49)^d^	0.04*
AUC_0–120min_	20,079 (3718)	20,590 (3762)	19,584 (4328)^c^	20,740 (3845)^d^	0.29
Tmax	51.0 (23.6)^a^	54.0 (22.6)^b^	56.9 (29.0)^c^	57.5 (23.2)^d^	0.62
Cmax	201 (37)	206 (46)	199 (43) ^c^	208 (42)^d^	0.65
Insulin, μU/ml	10.9 (5.6)	10.2 (4.7)	10.3 (5.0)	10.7 (5.9)	0.25
Glucagon, ρg/ml	185 (45)	189 (71)	183 (41)	187 (43)	0.50
HbA1c, %	5.70 (0.30)	5.69 (0.31)	5.68 (0.31)	5.69 (0.29)	0.61
C-peptide, ρg/ml	3.17 (1.09)	3.08 (1.06)	3.15 (1.09)	3.16 (1.41)	0.70
HOMA-IR	2.86 (1.52)	2.69 (1.41)	2.69 (1.44)^c^	2.82 (1.82)^d^	0.18
Triglyceride, mg/dL	143 (64)	141 (67)	142 (65)	138 (67)	0.86
Cholesterol, mg/dL	180 (45)	179 (40)	177 (36)	178 (36)	0.58
HDL-c, mg/dL	54.7 (13.8)	52.3 (11.6)	53.6 (11.8)	53.7 (11.4)	0.22
LDL-c, mg/dL	104 (39)	98.4 (34.4)	98.9 (33.5)	99.1 (31.7)	0.79
Non-HDL, mg/dL	127 (42)	124 (36)	123 (36)	123 (36)	0.76
Artery blood pressure					
Systolic, mmHg	127 (16)	125 (15)	122 (15)^c^	127 (17)^d^	0.02*
Diastolic, mmHg	80.7 (8.9)	80.3 (9.6)	78.6 (9.3)	79.4 (8.0)	0.43

OGTT (Oral Glucose Tolerance Test), HbA1c (Glycate hemoglobin), Non-HDL (LDL + VLDL + IDL + Lp (a)). Statistical intragroup differences between week 0 and week 12 are indicated by different letters (“a” vs. “b” for the citrus flavonoids group and “c” vs. “d” for the placebo group) (*p* ≤ 0.05). Intergroup differences between the citrus flavonoids and placebo groups are indicated by an asterisk (*) (*p* ≤ 0.05). Statistically significant differences are indicated by the values highlighted in bold.

**Figure 3:**
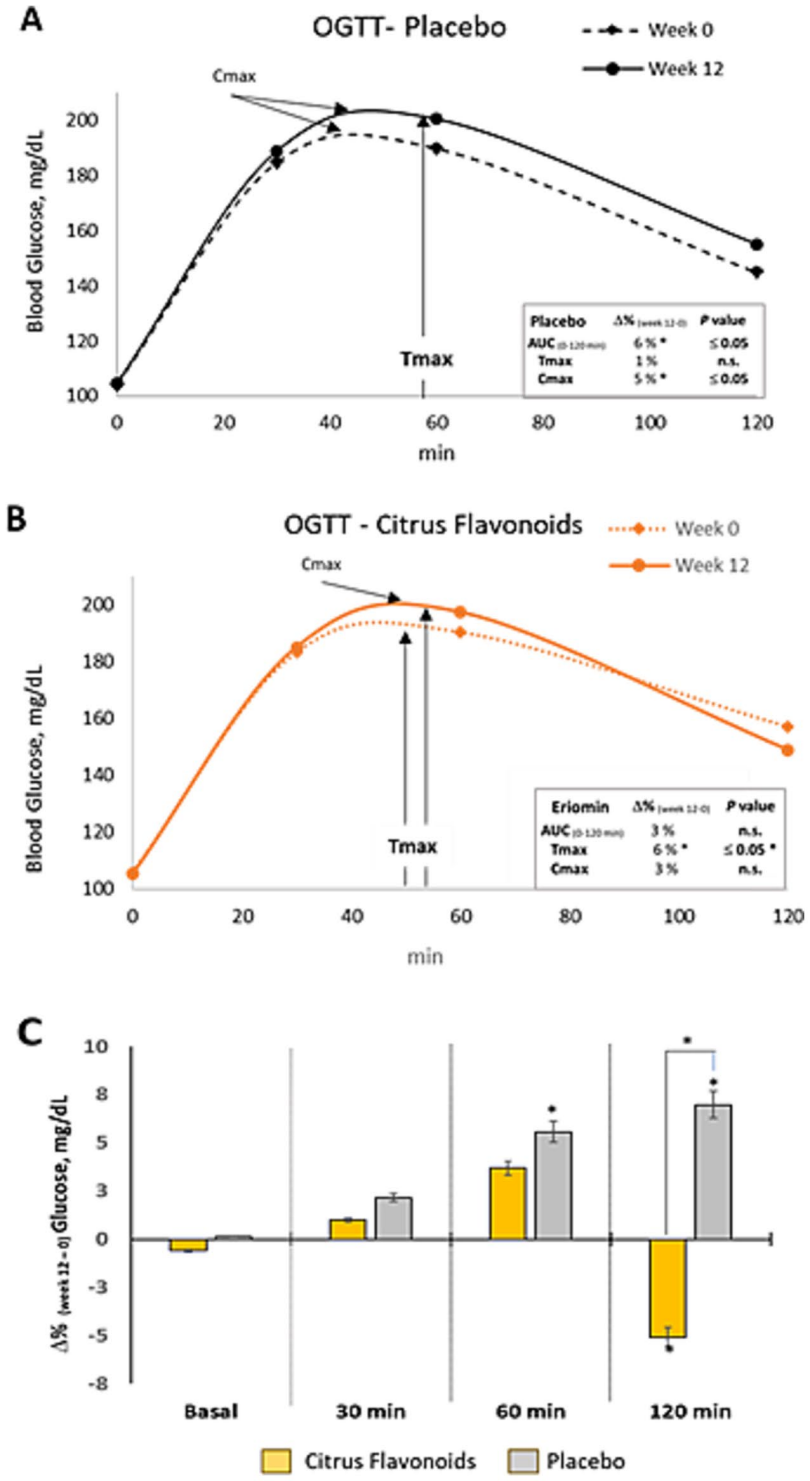
Oral glucose tolerance test (OGTT) in prediabetic patients treated with metformin plus citrus flavonoids or placebo. **(A)** Glucose clearance over 120 min following a 75 g oral dextrose load, measured at baseline and after 12 weeks of placebo treatment; **(B)** Glucose clearance at baseline and after 12 weeks of citrus flavonoids add-on treatment; **(C)** Intragroup differences between week 0 and week 12 are indicated by an asterisk (*) (*p* ≤ 0.05), and intergroup differences between the citrus flavonoids and placebo groups are indicated by a hash symbol (#) (*p* ≤ 0.04).

Glucose metabolism, assessed by the area under the curve (AUC₀–₁₂₀ min), peak glucose concentration (Cmax), and time to peak concentration (Tmax), showed distinct responses after 12 weeks. The Placebo group demonstrated a 6% increase in AUC, 4.5% increase in Cmax, and 1% increase in Tmax, indicating impaired glucose clearance. Conversely, the Citrus Flavonoids group exhibited a 6% increase in Tmax, while AUC and Cmax remained stable. These results suggest that Citrus Flavonoids may delay glucose absorption and enhance postprandial glucose regulation, which could be especially beneficial for individuals with prediabetes (Figure 3).

Although insulin, glucagon, HbA1c, and C-peptide levels remained comparable between groups (Table 3), insulin resistance, as measured by HOMA-IR, increased in the Placebo group but remained unchanged in the Citrus Flavonoids group, supporting a positive metabolic effect. These findings point to a synergistic benefit of key citrus flavonoids—eriocitrin, hesperidin, and naringin—on glucose regulation and insulin sensitivity.

Regarding biochemical markers, no significant changes were observed in fasting glucose, insulin, glucagon, HbA1c, C-peptide, triglycerides, total cholesterol, HDL-C, LDL-C, or non-HDL-C in either group after 12 weeks (Table 3). However, slight increases in glucose and insulin levels in the Placebo group contributed to a significantly higher HOMA-IR, further reinforcing the beneficial metabolic impact of the citrus flavonoid supplementation.

Systolic and diastolic blood pressure were also evaluated in both groups. Systolic pressure was significantly higher in the Placebo compared to the Citrus Flavonoids treatment. When comparing the two treatment periods within the same individuals (due to the crossover design), systolic pressure improved significantly during the Citrus Flavonoid supplementation phase. These results suggest that non-supplemented patients may benefit from flavonoid treatment, as the observed blood pressure improvements appear to be associated with the bioactive properties of the citrus flavonoids.

### Organ function biomarkers

At week 12, liver, pancreatic, and kidney function markers were assessed (Table 4). The Aspartate Aminotransferase (AST) levels were significantly higher in the placebo group, while pancreatic amylase and serum creatinine were significantly lower in the citrus flavonoid group (*p* ≤ 0.01). No other significant changes in liver, pancreatic, or renal biomarkers were observed between or within groups over the 12-week period.

**Table 4:** Characteristics of liver and urine biochemicals of prediabetic patients treated with metformin combined with citrus flavonoids or placebo for 12 weeks.

Treatment with metformin add-on	Citrus flavonoids (*n* = 68)		Placebo (*n* = 68)		*P* value
	Week 0	Week12	Week 0	Week12	
	Mean (SD)	Mean (SD)	Mean (SD)	Mean (SD)	
Aspartate aminotransf., U/L	22.6 (9.7)	20.1 (7.8)	21.2 (5.9)^c^	24.4 (9.5)^d^	<0.01*
Alanine aminotransf., U/L	29.6 (20.7)	26.5 (15.5)	27.9 (15.5)	27.1 (16.9)	0.58
γ-glutamyl transferase, U/L	36.9 (32.9)	33.6 (21.3)	34.9 (23.4)	31.8 (15.5)	0.77
Alkaline phosphatase, U/L	64.9 (19.5)	63.2 (19.3)	62.9 (17.6)	61.4 (19.3)	0.72
Amylase, mg/dL	80.8 (33.1)^a^	72.6 (30.3)^b^	74.6 (32.3)	71.7 (31.3)	0.05*
Lipase, mg/dL	35.3 (19.9)	30.4 (17.3)	37.4 (33.8)	31.7 (20.1)	0.72
Urea, mg/dL	33.3 (11.7)	32.4 (10.5)	34.2 (10.8)	35.3 (14.2)	0.32
Creatinine, mg/dL	0.82 (0.18)^a^	0.75 (0.17)^b^	0.87 (0.24)	0.86 (0.3)	0.08
Uric Acid	4.60 (1.13)	4.47 (1.10)	4.60 (1.05)	4.45 (1.00)	0.83

Alanine Aminotransferase (ALT); Aspartate Aminotransferase (AST); Gamma-Glutamyl Transferase (GGT); Alkaline Phosphatase (ALP). Statistical intragroup differences between week 0 and week 12 are indicated by different letters (“a” vs. “b” for the citrus flavonoids group and “c” vs. “d” for the Placebo group) (*p* ≤ 0.05). Intergroup differences between the citrus flavonoids and placebo groups are indicated by an asterisk (*) (*p* ≤ 0.05). Statistically significant differences are indicated by the values highlighted in bold.

### Peptides, hormones, and inflammatory markers

Active GLP-1 levels decreased by 15% in the Placebo group, while remaining stable in the Citrus Flavonoids group (Table 5). Secretin levels increased by 7% in the Placebo group but remained unchanged in the Citrus Flavonoid group (*p* ≤ 0.03). Conversely, peptide YY (PYY) levels decreased by 12% in the Citrus Flavonoid group, representing a statistically significant reduction compared to the Placebo group (*p* < 0.02). No significant differences were observed between groups for other metabolic hormones or peptides.

**Table 5:** Evaluation of peptides, hormones, inflammatory markers, and oxidative stress in prediabetic patients treated with metformin combined with citrus flavonoids or placebo for 12 weeks.

Treatment with metformin add-on	Citrus flavonoids (*n* = 68)		Placebo (*n* = 68)		*P* value
	Week 0	Week12	Week 0	Week12	
	Mean (SD)	Mean (SD)	Mean (SD)	Mean (SD)	
Peptides and hormones					
Total GLP-1, ρmol/L	11.2 (5.5)	11.6 (6.3)	12.0 (6.5)	11.4 (5.7)	0.54
Active GLP-1, ρmol/L	5.9 (4.5)	5.7 (4.1)	6.5 (5.2)^c^	5.5 (4.1)^d^	0.14
GIP, ρmol/L	32.7 (19.5)	29.7 (14.5)	30.3 (17.7)	30.9 (14.1)	0.21
Total amylin, ρmol/L	13.8 (10.0)	12.0 (8.7)	11.9 (8.2)	11.7 (8.2)	0.49
Ghrelin, ρmol/L	20.2 (14.8)	21.5 (16.3)	22.5 (20.8)	22.2 (13.8)	0.36
Secretin, ρmol/L	22.6 (28.5)	23.3 (29.6)	24.6 (37.0)^c^	26.4 (36.7)^d^	**0.03**
Peptide YY, ρmol/L	54.8 (16.7)^a^	48.3 (15.5)^b^	45.5 (10.0)	47.9 (12.6)	**0.02**
Pancreatic P, ρmol/L	38.3 (26.9)	39.8 (27.6)	38.8 (29.1)	39.5 (28.3)	0.97
Leptin, ng/mL	37.8 (26.2)	37.3 (27.5)	37.5 (25.4)	37.9 (27.4)	0.60
Inflammatory and oxidative stress					
Interleukin-6, ρg/mL	9.27 (6.97)	8.15 (5.28)	8.23 (6.13)	8.91 (7.39)	0.29
TNF-α, ρg/mL	15.2 (5.0)^a^	14.8 (4.5)^b^	15.4 (5.7)	15.3 (5.2)	0.27
MCP-1, ρg/mL	254 (101)	240 (96)	256 (104)	239 (83)	0.98
CRP, mg/dL	0.44 (0.49)	0.41 (0.45)	0.38 (0.40)	0.39 (0.48)	0.75
FRAPE, μM	1242 (289)^a^	1334 (539)^b^	1254 (415)^c^	1090 (274)^d^	**0.05**

Intragroup differences between week 0 and week 12 are indicated by “a ≠ b” for the citrus flavonoids group and “c ≠ d” for the placebo group (*p* ≤ 0.05). Intergroup differences between the citrus flavonoids and placebo groups are indicated by “*” (*p* ≤ 0.05). Statistically significant differences are indicated by the values highlighted in bold.

Among the inflammatory and oxidative stress biomarkers, TNF-*α* levels decreased by 12% and FRAP (ferric reducing ability of plasma) increased by 7.5% in the Citrus Flavonoids group. In contrast, FRAP levels decreased by 13% in the Placebo group. These changes indicate a significant antioxidant and anti-inflammatory benefit associated with Citrus Flavonoid supplementation in prediabetic individuals. All other biomarkers remained unchanged in both treatment arms (Table 5).

### Tolerability and nutrient intake

Citrus flavonoid supplement was well tolerated and showed a similar safety profile to placebo. Nine gastrointestinal adverse events were reported, mostly among participants newly initiated on metformin (*n* = 48). Three participants were withdrawn due to persistent symptoms exceeding 1 week. Baseline characteristics were statistically similar between groups (*p* > 0.05; Table 1).

## Discussion

Metformin therapy in individuals with prediabetes reduces the risk of progression to type 2 diabetes by 7.2% (absolute risk reduction; *p* ≤ 0.001) and promotes modest weight loss (19). Mechanistically, metformin primarily activates AMP-activated protein kinase (AMPK) by inhibiting complex I of the mitochondrial respiratory chain, thereby increasing the AMP: ATP ratio. This metabolic shift stimulates fatty acid oxidation, enhances glucose uptake, and downregulates genes involved in lipogenesis and gluconeogenesis. Moreover, AMPK-mediated phosphorylation of acetyl-CoA carboxylase (ACC) further contributes to metformin’s lipid-lowering and insulin-sensitizing actions (20).

Beyond its established glucose-lowering effects, metformin has also been shown to enhance GLP-1 secretion, potentially by delaying carbohydrate absorption and modulating DPP-4 activity, contributing to improved incretin responses in patients with type 2 diabetes (21). Additionally, metformin exerts anti-inflammatory effects via AMPK and NF-κB inhibition, leading to reductions in IL-6, TNF-*α*, and CRP levels (22), and provides indirect antioxidant benefits through improved redox balance (23). However, gastrointestinal adverse effects—primarily diarrhea and nausea—occur in up to 25% of patients. Since the glucose-lowering effect of metformin is dose-dependent, initiating treatment at low doses and gradually titrating upward, along with the use of extended-release formulations, may improve tolerability (23).

Recent studies have demonstrated that combining metformin therapy with natural compounds exhibiting antidiabetic activity may enable effective glycemic control with lower doses of metformin, thereby reducing side effects while enhancing clinical outcomes. For instance, co-administration of berberine with metformin significantly reduced HbA1c and fasting blood glucose (FBG) levels compared to metformin alone, with fewer gastrointestinal side effects (14, 24). Berberine acts primarily via AMPK activation, leading to improved insulin sensitivity, reduced hepatic gluconeogenesis, and enhanced peripheral glucose uptake. It also inhibits intestinal *α*-glucosidase, reducing postprandial glucose excursions, and exerts anti-inflammatory and antioxidant effects by modulating NF-κB and upregulating Nrf2 and SOD (25, 26).

Citrus flavonoids have been widely studied for their ability to stimulate GLP-1 secretion, thereby enhancing insulin release and supporting glucose homeostasis. Hesperidin activates the TGR5 receptor in intestinal L cells, triggering the cAMP-CREB pathway to promote GLP-1 release (27). Naringin, on the other hand, inhibits dipeptidyl peptidase-4 (DPP-4), the enzyme responsible for GLP-1 degradation, thereby prolonging GLP-1 activity (28). Eriocitrin, a key component of citrus flavonoid nutraceutical, has been shown to increase GLP-1 levels by 17% in prediabetic and diabetic patients after 12 weeks of supplementation (7) and to reduce inflammatory cytokines such as IL-6 and TNF-α, improving HOMA-IR and other metabolic biomarkers (6).

To clarify that the significant improvements observed—especially in markers like HbA1c, GLP-1, and inflammatory biomarkers—should be interpreted in the context of the supplement’s preventive and supportive role, and not as a substitute for pharmacological treatment. The safety profile and tolerability of citrus flavonoids also support their potential value in long-term metabolic health management. Also, although the crossover design of this study reduced inter-individual variability and strengthened the detection of treatment effects, we acknowledge that participant characteristics such as sex, age, and ethnicity may influence responses to citrus flavonoid supplementation. Due to the relatively small sample size, we were not able to conduct stratified subgroup analyses to explore these effects. Future clinical trials with larger and more diverse populations are warranted to examine whether demographic variables modulate the metabolic and glycemic responses to citrus bioflavonoids, particularly in real-world, heterogeneous patient populations.

In the present study, the addition of the supplement to metformin monotherapy in prediabetic patients enhanced metabolic outcomes beyond those achieved with metformin alone, improving insulin sensitivity (HOMA-IR) and glucose clearance (AUC₀_−_₁₂₀ mg·min/dL). In contrast, during the placebo add-on phase, no significant changes were observed in fasting glucose or at 30 min following the oral glucose load. Sustained active GLP-1 levels during citrus flavonoid supplementation further support its potential to provide additive metabolic benefits when combined with metformin.

Among natural compounds, the alkaloid berberine has been shown to increase GLP-1 levels, potentially through modulation of the gut microbiota and stimulation of intestinal L-cells (26). The flavonoid hesperidin significantly enhances GLP-1 secretion and reduces pro-inflammatory biomarkers such as IL-6, TNF-*α*, and hsCRP in both clinical and preclinical models of non-alcoholic fatty liver disease (27). Similarly, naringin exhibits comparable effects, decreasing inflammatory cytokines and enhancing antioxidant enzyme activity (28, 29). Eriocitrin, a key component of citrus flavonoids, has also been shown to reduce oxidative stress and support metabolic improvements in prediabetic populations (30). Although quercetin, epigallocatechin gallate (EGCG), and curcumin do not directly stimulate GLP-1 secretion, they exert potent anti-inflammatory and antioxidant effects primarily through NF-κB inhibition and Nrf2 activation (31). Taken together, metformin and berberine remain the most potent AMPK activators among the compounds discussed, while hesperidin, naringin, and eriocitrin provide moderate AMPK activation and complement metabolic benefits through GLP-1 upregulation, anti-inflammatory effects, and antioxidant activity. Notably, metformin, berberine, and citrus flavonoids all contribute to enhanced GLP-1 secretion or signaling, primarily through mechanisms involving L-cell activation and gut microbiota modulation.

Although previous studies reported a significant increase in GLP-1 levels with citrus flavonoid supplementation, the current study—where patients received it daily as an add-on to metformin—did not show a change in total GLP-1 levels. However, active GLP-1 levels decreased significantly in the placebo group but remained stable in the supplemented group, suggesting that it may inhibit DPP-4 activity and preserve active GLP-1. This finding aligns with known mechanisms by which prolongation of GLP-1 activity contributes to improved glycemic control. It is important to note that total GLP-1 reflects both the active and inactive forms, with the latter rapidly degraded by DPP-4 shortly after secretion (32). Therefore, distinguishing between active and total GLP-1 is critical for understanding the role of citrus flavonoids as an adjunct to metformin therapy.

Metformin has been reported to reduce amylin secretion, possibly as a result of improved insulin sensitivity and reduced pancreatic *β*-cell stress (33). However, in the present study, no significant differences in amylin biomarker were observed with metformin plus citrus flavonoids or placebo. Although elevated amylin levels may aid in glycemic control, excessive accumulation is associated with pancreatic amyloid deposits—a pathological feature of type 2 diabetes. Thus, metformin helps maintain amylin within physiological levels, alleviating β-cell stress and reducing the risk of amyloid formation (34), while citrus flavonoids add-on therapy did not affect this metabolic pathway.

Other gastrointestinal hormones, including ghrelin, pancreatic polypeptide (PP), and leptin, were not significantly affected by supplementation. Interestingly, peptide YY (PYY) levels were lower in the citrus flavonoids group compared with placebo. A reduction in PYY may reflect decreased hormonal signaling or altered nutrient response, potentially linked to metabolic dysfunctions such as obesity and prediabetes (35)—conditions present in the majority (64%) of patients in this study.

Additionally, secretin levels increased in the metformin plus placebo group but remained stable in the supplemented group. Secretin, produced by duodenal S cells, stimulates pancreatic secretion of bicarbonate to neutralize gastric acid in the small intestine. Elevated secretin levels may indicate the presence of excess acidic chyme in the duodenum or gastrointestinal disorders such as inflammation or dysregulated acid secretion (36). These findings suggest possible differences in gastrointestinal physiology between the groups, although such parameters were not directly assessed.

Regarding lipid metabolism, no improvements in lipid profiles were observed in either the citrus flavonoids or placebo add-on groups during the treatment period. However, given that the study population was relatively young and in the early stages of prediabetes, baseline cardiovascular risk was likely low. It is well established that both diabetes and dyslipidemia are independent risk factors for atherosclerotic cardiovascular disease (CVD), and that dyslipidemia plays a central role in macrovascular complications. Furthermore, the coexistence of insulin resistance and dyslipidemia further elevates myocardial infarction risk (37). Thus, by improving insulin sensitivity, the addition of citrus flavonoid supplement to metformin therapy may help reduce long-term CVD risk in prediabetic individuals.

In both preclinical and clinical studies, citrus flavonoids supplementation has shown a favorable safety profile regarding pancreatic and renal biomarkers (6, 7). In rodent models, a slight early increase in serum amylase has been observed, likely reflecting adaptive stimulation of digestive enzyme secretion rather than pancreatic injury (38). Mild elevations in creatinine have also been reported, interpreted as an effect of enhanced muscle metabolism through AMPK activation rather than renal dysfunction (39, 40). Clinical trials in prediabetic patients confirmed these findings, showing no significant alterations in creatinine levels after 12 weeks of supplementation at doses up to 800 mg/day (6, 7). Furthermore, citrus flavonoids demonstrated protective effects against oxidative stress and systemic inflammation, supporting its metabolic benefits without compromising pancreatic or renal function.

Other notable changes in the metabolic profile of prediabetic patients treated with citrus flavonoids included significant reductions in body weight, fat mass, and BMI. These improvements are likely associated with enhanced glycemic control and stabilization of active GLP-1 levels. In contrast, the placebo group exhibited a decrease in GLP-1, which may have contributed to impaired glucose clearance. Additionally, the supplemented group showed a reduction in interleukin-6 (IL-6) levels and an increase in antioxidant capacity (as measured by FRAP), whereas the placebo group experienced a decline in FRAP. These anti-inflammatory and antioxidant effects likely contributed to the improved metabolic outcomes observed with supplementation. Furthermore, systolic blood pressure decreased significantly in the citrus flavonoids group, while it increased in the placebo group. This effect may be related to the modulation of IL-6 levels, improved antioxidant status, and the higher dietary potassium intake observed in the supplemented group. Taken together, these findings provide valuable insights into how citrus flavonoids may enhance the metabolic benefits of metformin. Nonetheless, additional *in vitro* and *in vivo* studies are necessary to further elucidate the mechanisms by which citrus flavonoids modulate gastrointestinal hormones, inflammatory biomarkers, and metabolic pathways in the context of metformin therapy.

Emerging evidence underscores the gut microbiota’s role in mediating the metabolic effects of both metformin and dietary polyphenols, including citrus flavonoids. These compounds are biotransformed by gut microbes into bioactive metabolites that support glycemic and lipid regulation. In our previous study (18), we observed that while citrus flavonoids alone induced modest microbial shifts, their combination with metformin significantly altered beta diversity and enhanced short-chain fatty acid (SCFA) production. Notably, the co-administration increased the abundance of butyrate-producing genera such as *Bifidobacterium* and *Collinsella*, along with higher levels of butyric and acetic acids—metabolites associated with improved insulin sensitivity and gut barrier function. We also observed a dose-sensitive modulation of *Coriobacteriaceae*, a family linked to polyphenol metabolism and metabolic health, which remained stable only in the high-dose combination group. These findings suggest a synergistic interaction between metformin and citrus flavonoids in shaping the gut microbiota and enhancing SCFA-mediated benefits, thereby offering greater metabolic improvements in prediabetic individuals than metformin alone.

This study has several strengths. It employed a randomized, double-blind, placebo-controlled design, providing robust evidence of the adjunctive effects of citrus flavonoids supplement with metformin. Comprehensive metabolic profiling—including glycemic, inflammatory, hormonal, and anthropometric markers—allowed for an in-depth evaluation of clinical outcomes. The 12-week duration was sufficient to capture early metabolic changes associated with prediabetes management. However, certain limitations should be acknowledged, including the relatively small sample size and the short follow-up period, which may limit the generalizability and long-term interpretation of the findings. Additionally, because the volunteers were relatively young and at low cardiovascular risk, no pronounced changes in lipid profiles or other lipid-correlated biomarkers were observed. Future studies with longer durations, larger and more diverse cohorts, and comprehensive gut microbiota assessments are warranted to further elucidate the long-term metabolic effects, potential mechanisms of action—particularly involving GLP-1 and DPP-4 pathways—and the broader applicability of citrus flavonoid-based nutraceutical in populations at higher risk for type 2 diabetes and cardiometabolic disorders.

In conclusion, citrus flavonoids supplementation as an adjunct to metformin therapy significantly improved postprandial glucose metabolism, preserved active GLP-1 levels, and reduced insulin resistance in prediabetic patients. Additionally, it exerted anti-inflammatory and antioxidant effects and modestly improved anthropometric and blood pressure parameters, which were reflected in favorable changes in several metabolic and inflammatory biomarkers. These biomarker improvements suggest that citrus flavonoids may enhance the metabolic efficacy of metformin and offer a safe, nutritional strategy for early intervention in prediabetes management. Further long-term studies are warranted to confirm these biomarker-related benefits and explore underlying mechanisms.

## Data Availability

The raw data supporting the conclusions of this article will be made available by the authors, without undue reservation.
